# Traditional Chinese medicine as a novel therapy for colorectal cancer by modulating intestinal flora

**DOI:** 10.7150/jca.87719

**Published:** 2023-09-04

**Authors:** Yushan Dong, Jingyu Chen, Heng Sun, Yuhan Chen, Yan Jiao, Weikuan Gu, Hong Chen, Songjiang Liu

**Affiliations:** 1 Graduate School of Heilongjiang University of Chinese Medicine, No. 24, Heping Road, Xiangfang District, Harbin, Heilongjiang, China, 150040.; 2 Department of Chinese Medicine Internal Medicine, Xiyuan Hospital, China Academy of Chinese Medical Sciences, No. 1, Xiyuan Playground, Haidian District, Beijing, 100091.; 3 Department of Chinese Medicine, The First Affiliated Hospital of Heilongjiang University of Chinese Medicine No.26, Heping Road, Xiangfang District, Harbin, Heilongjiang Province, China, 150040.; 4 Department of Chinese Medicine, Guang'anmen Hospital, China Academy of Chinese Medical Sciences, Beijing, China, 100091.; 5 Department of Chinese Medicine, Heilongjiang Academy of Chinese Medicine Sciences, No. 72 Xiangan Street, Xiangfang District, Harbin, China, 150036.; 6 Department of Orthopedic Surgery and BME, College of Medicine, University of Tennessee Health Science Center, Memphis, TN, 38163, USA.

**Keywords:** Bifidobacterium bifidum, Colorectal cancer, Traditional Chinese Medicine, Intestinal flora

## Abstract

Colorectal cancer is a common clinical malignant tumor of the digestive tract, and intestinal flora has played an important role in the development of colorectal cancer. Bifidobacteria, as one of the main dominant florae in intestinal tract, can inhibit the occurrence and development of colorectal cancer through various mechanisms. Recent studies have shown that traditional Chinese medicine can regulate the abundance of bifidobacteria in intestinal tract and exhibit anti-tumor effects on colorectal cancer. Detailed investigations have revealed that the mechanisms of bifidobacteria in the treatment of colorectal cancer involve three aspects: the production of short-chain fatty acids, the regulation of the body's immunity, and the regulation of cell apoptosis and differentiation. In this review, we provide an updated summary of recent advances in our understanding of the mechanisms by which traditional Chinese medicine regulate intestinal flora to inhibit colorectal cancer development and metastasis.

## Introduction

Colorectal cancer is one of common clinical malignant tumors of the digestive tract 1. Recently, with the changes in lifestyles and diets, the incidence and mortality of colorectal cancer have shown an upward trend 2. Worldwide incidence and mortality of colorectal cancer have been ranked the third and the second, respectively 3. At present, clinical treatments of colorectal cancer mainly adopt surgery, radiotherapy and chemotherapy, and targeted therapy. It has been confirmed that traditional Chinese medicine can effectively reduce the side effects of these treatments, increase the patient's tolerance to treatment, enhance the immune function of the body, prolong the survival of patients, improve the quality of life, and inhibit tumor metastasis and recurrence 4.

Recent studies suggest that traditional Chinese medicine can have therapeutic effect on colorectal cancer by modulating intestinal flora 5. Therefore, it is important to understand how traditional Chinese medicine regulate intestinal flora to promote the prevention and treatment of colorectal cancer. In this review, we summarize recent advances in the investigations of the relationship between traditional Chinese medicine, intestinal flora and colorectal cancer to provide novel strategies for the prevention and treatment of colorectal cancer.

## Side effects of current treatment methods of colorectal cancer

**Figure 1:**
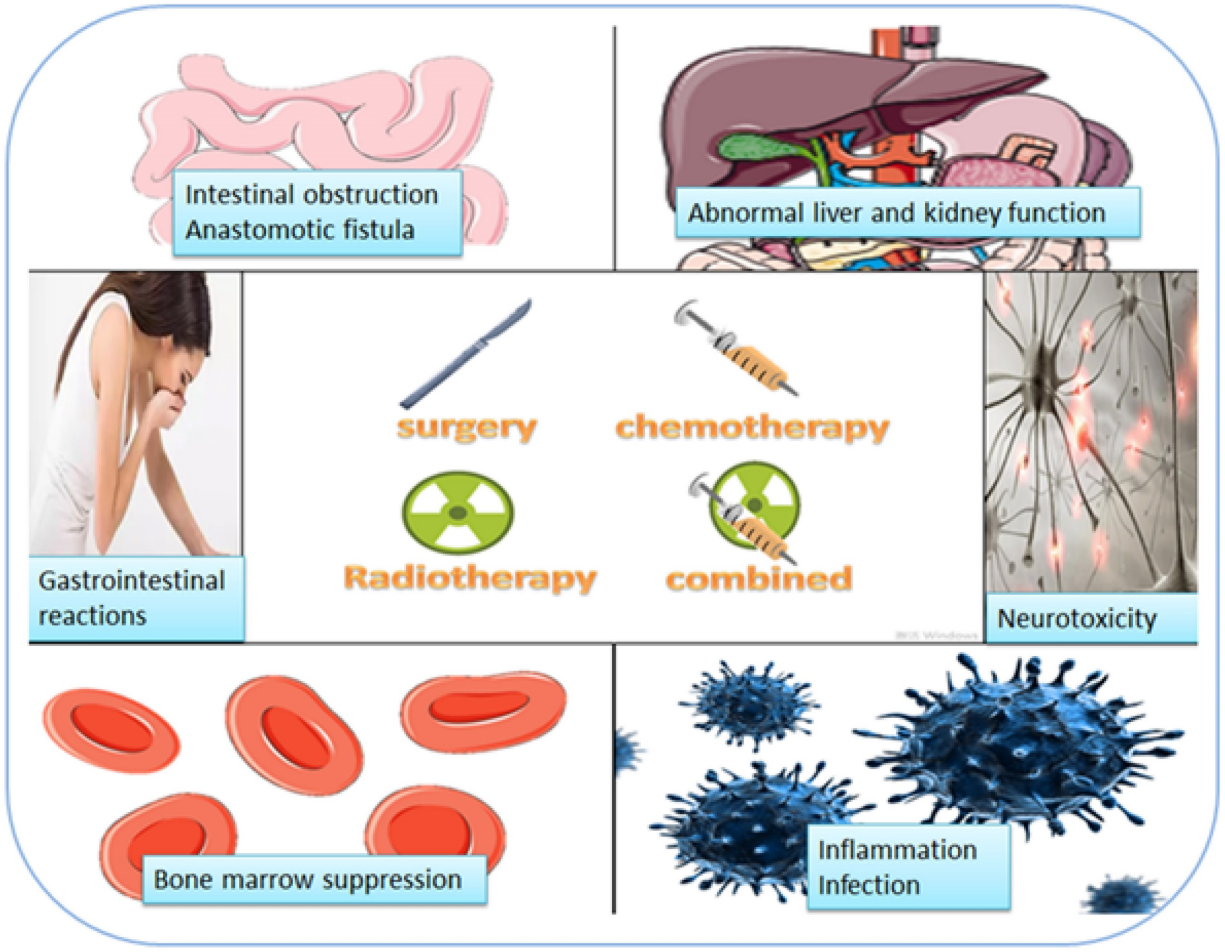
** Side effects of treatment of colorectal cancer by surgery, radiotherapy and chemotherapy.** Common clinical complications of surgery for colorectal cancer include incision infection, fever, urinary tract infection, abdominal infection, intestinal obstruction, anastomotic leakage, etc. Chemotherapy causes side effects including pancytopenia, gastrointestinal reactions such as nausea and vomiting, diarrhea, loss of appetite, abnormal liver and kidney function and neurotoxicity. Adverse reactions of radiation therapy mainly include radiation proctitis, radiation cystitis, skin injury, intestinal adhesion, intestinal obstruction, incision infection, urinary tract infection, lower extremity edema. The main adverse reactions of combined radiotherapy and chemotherapy include bone marrow suppression, hand-foot syndrome, neurotoxicity, gastrointestinal reactions (nausea, vomiting, diarrhea), and phlebitis.

Current treatment methods of colorectal cancer mainly include surgery, radiotherapy, chemotherapy and combined treatment. Unfortunately, these treatment methods have a variety of side effects as summarized in Figure 1.

## Side effects of surgery

At present, clinical treatment of colorectal cancer is mainly based on surgery. Because laparoscopic surgery has the advantages of small incision, less blood loss, and rapid postoperative recovery, it has been promoted and popularized clinically, and become the main surgical method at present. Common clinical complications of laparoscopic surgery for colorectal cancer include incision infection, fever, urinary tract infection, abdominal infection, intestinal obstruction, anastomotic leakage, etc. These complications adversely affect postoperative recovery of the patients and lead to poor prognosis 6.

## Side effect of chemotherapy

Chemotherapy after surgery for colorectal cancer patients can significantly improve postoperative survival rate and reduce the probability of recurrence and metastasis. However, chemotherapy drugs have certain toxicity, and patients experience a series of side effects including pancytopenia, gastrointestinal reactions such as nausea and vomiting, diarrhea, loss of appetite, abnormal liver and kidney function and neurotoxicity 7. These side effects affect the prognosis of the patients and even lead to the forced discontinuation of chemotherapy.

## Side effect of radiotherapy

Radiation therapy plays an important role in the treatment of colorectal cancer. Transient complications often occur during radiotherapy, which does not have a significant impact on the overall treatment, but a small number of patients may still experience serious complications, leading to discontinuation of treatment. Adverse reactions of radiation therapy mainly include radiation proctitis, radiation cystitis, skin injury, intestinal adhesion, intestinal obstruction, incision infection, urinary tract infection, lower extremity edema. 8

## Side effect of combined radiotherapy and chemotherapy

In recent years, studies have shown that although a single surgical treatment or radiotherapy and chemotherapy have good clinical effects, their efficacy still needs to be improved, and the simultaneous application of radiotherapy and chemotherapy can significantly improve the survival of patients. At present, the main adverse reactions of combined radiotherapy and chemotherapy include bone marrow suppression, hand-foot syndrome, neurotoxicity, gastrointestinal reactions (nausea, vomiting, diarrhea), and phlebitis 9.

## Intestinal flora

**Figure 2:**
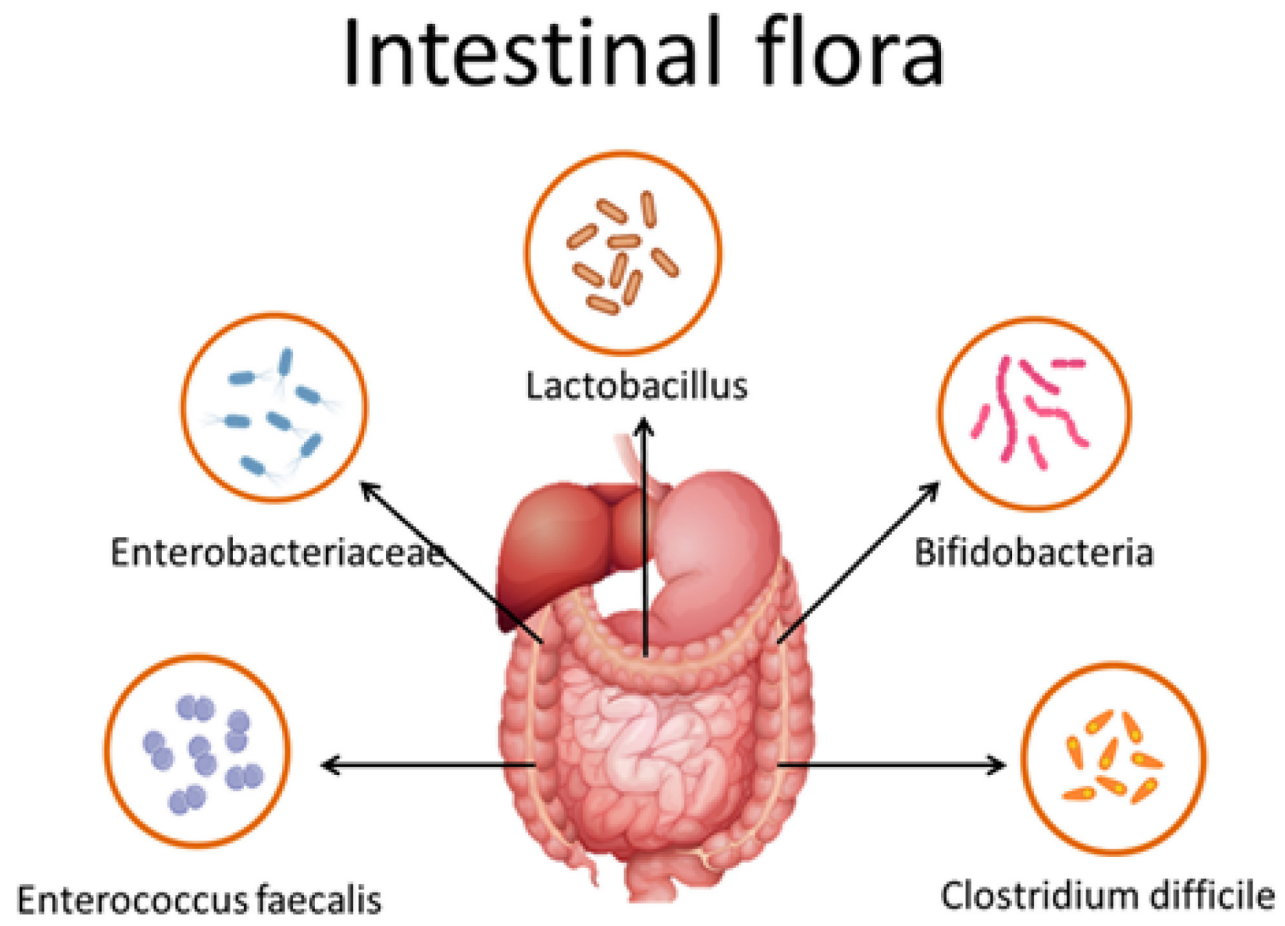
** Intestinal flora carried by the host for life.** The main bacteria in the intestine include Bifidobacteria, Lactobacillus, Enterobacteriaceae, Enterococcus faecalis and Clostridium difficile.

In the gut, there are a wide variety of bacteria and their roles are diverse. Bifidobacteria have a huge number in the gut flora and interacts with other bacteria in the gut to keep intestinal flora (Figure 2). Intestinal flora could maintain normal structure and physiological functions of the intestinal tract, antagonize the colonization of pathogenic microorganisms, and regulate the immune function of the human body 10.

Bifidobacterium is a Gram-positive polymorphic bacillus, and its main functions are to antagonize the colonization of harmful bacteria, improve the metabolism of vitamins, promote gastrointestinal motility, improve liver function, and produce immune factors to enhance human immune function. As one of the main probiotics in the intestinal flora, bifidobacteria could be utilized in the prevention and treatment of colorectal cancer. 11.

## The mechanism of action of bifidobacteria in the treatment of colorectal cancer

### Production of short-chain fatty acids

Short-chain fatty acids (SCFA) are the final products of dietary fiber and carbohydrates metabolized in the intestinal tract, which mainly include acetate, propionate, and butyrate. SCFA can effectively regulate the inflammatory response of the intestinal mucosa, inhibit the growth of related pathogens, affect intestinal peristalsis, promote the apoptosis of cancer cells and inhibit the differentiation of cancer cells 12. Bifidobacteria significantly increased the content of butyric acid, isobutyric acid and other SCFAs in the gastrointestinal tract, and inhibited the growth of tumor cells by promoting the production of SCFAs 13. Bifidobacteria can also produce lactic acid and acetic acid to acidify the intestinal tract to accelerate peristalsis, reduce the contact between carcinogens and intestinal mucosal epithelium, and inhibit the proliferation and differentiation of cancer cells. Therefore, bifidobacteria generate SCFAs through metabolism, inhibits the growth and differentiation of cancer cells, promotes the apoptosis of cancer cells, and plays a role in the prevention and treatment of colorectal cancer.

### Enhancement of immunity

The body's immune system could fight against cancer by affecting immune cells such as macrophages, T cells, and natural killer cells (NK) 14, 15. Bifidobacteria may inhibit the occurrence and development of colorectal cancer by regulating these immune cells. Bifidobacteria can induce the activation of macrophages in the host, promote the secretion of tumor necrosis factor (TNF-α) by macrophages, and inhibit the proliferation of cancer cells 16. Bifidobacteria also activate NK cells, LAK cells, CD8+T, CD4+ and other immune cells, and promote the production of cytotoxic factors to kill cancer cells 17,18. The intact peptidoglycan (WPG) of bifidobacteria can activate macrophages and promote macrophages to secrete IL-1, IL-6, TNF-α to inhibit tumor cells 19. Bifidobacteria can promote the apoptosis of tumor cells and inhibit their differentiation and proliferation by inducing the activation of immune cells and promoting their secretion of tumor-killing effectors.

### Regulation of apoptosis and cell proliferation

**Figure 3:**
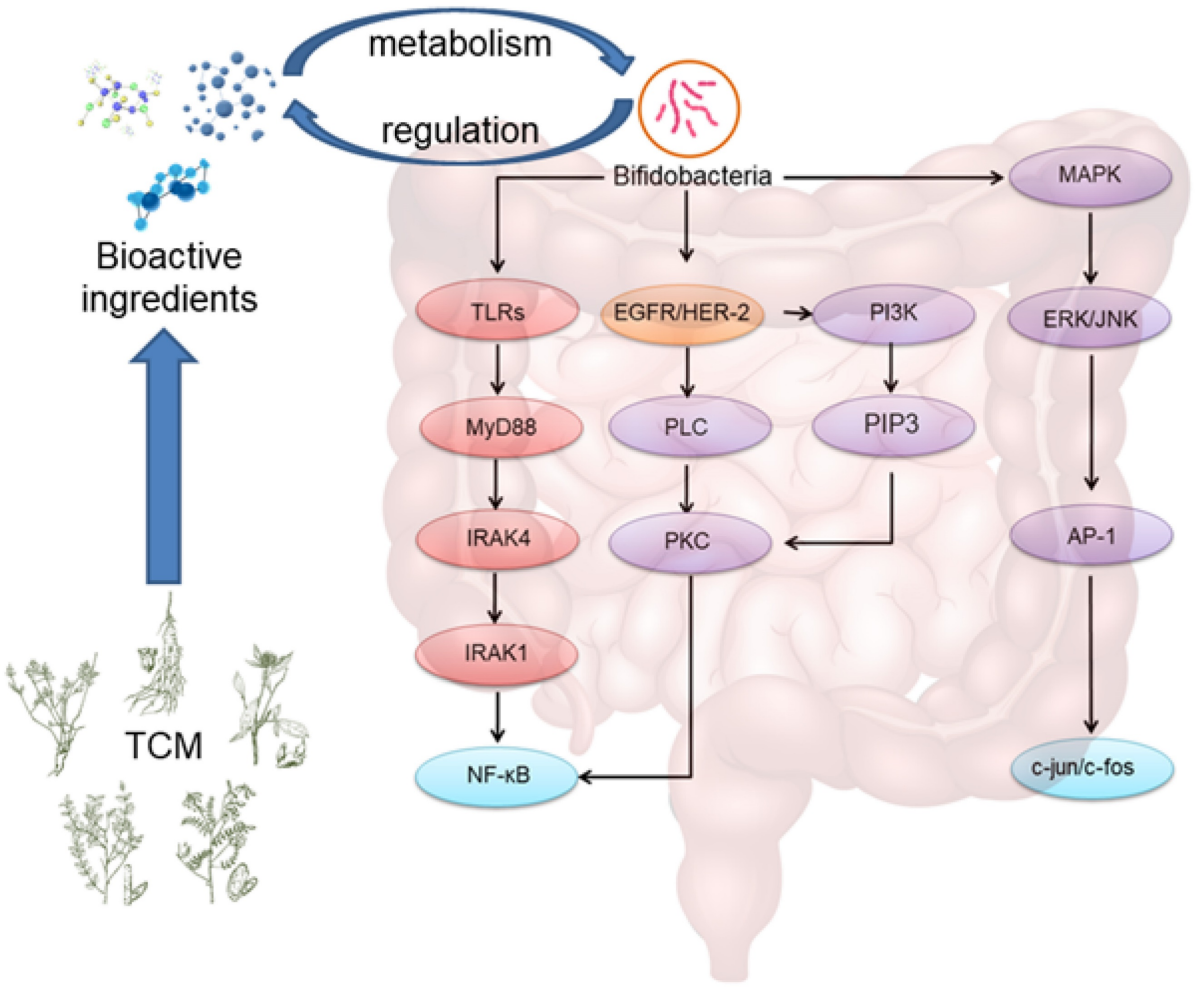
** Molecular pathways of the mechanism of action of bifidobacteria in intestinal flora.** When TCM enter the intestine, bifidobacteria metabolize TCM to increase the efficacy or reduce the toxicity of bioactive ingredients of TCM. On the other hand, bioactive ingredients of TCM can regulate the balance of bifidobacteria.

The second messengers in the cells include cAMP, cGMP, inositol triphosphate (IP3), diacylglycerol (DAG) (Figure 3). Recent studies have shown that bifidobacteria can fight tumors by regulating second messengers, affecting their signal transduction, and regulating cell activities 20. Faghfoori et al. found that the supernatant of bifidobacteria significantly upregulated the expression of bad, caspase-8, and Fas-R in cells, suggesting that bifidobacteria can promote the expression of pro-apoptotic genes 21.

Bifidobacteria decreased the expression of EGFR and HER-2 in colon cancer cells, and promoted the apoptosis of colon cancer cells 22.

## The interaction between traditional Chinese medicine and intestinal flora

Traditional Chinese medicine is usually administrated by oral route. Once traditional Chinese medicine enters digestive tract, it regulates microenvironment and maintain the balance of intestinal flora. Conversely, intestinal flora promotes the transformation and metabolism of traditional Chinese medicine (Figure 3) 23. The special types of traditional Chinese medicine and their interaction with intestinal flora have been extensively reviewed in recent literatures 24, 25. Since intestinal flora plays an important role in the development of colorectal cancer, it is expected that traditional Chinese medicine could provide novel approach for the prevention and treatment of colorectal cancer by regulating the balance of intestinal flora 26.

## Conclusions

The imbalance of intestinal microecology is related to the occurrence and development of digestive system diseases such as colorectal cancer. In recent years, the role of intestinal flora in colorectal cancer has been paid more attention and the modulation of intestinal flora has become a novel strategy for the treatment of colorectal cancer.

The mechanism by which microflora inhibits cancer cells is mainly to promote the production of SCFA, regulate the body's autoimmunity, affect cell signaling pathway, and change the expression of colorectal cancer-related factors 27. The application of traditional Chinese medicine can promote the abundance of bifidobacteria and has a significant therapeutic effect on colorectal cancer. Therefore, traditional Chinese medicine may have therapeutic effect on colorectal cancer by regulating bifidobacteria, but the underlying mechanisms need further investigations.

## Challenges

With the development of traditional Chinese medicine, good anti-tumor effects have been achieved. However, traditional Chinese medicine is not composed of a single component but has extremely complex chemical components, which leads to the uncertainty of its application for the treatment of colorectal cancer. In addition, some patients have intolerance to the special ingredients, which in turn produces allergic reactions and affects the therapeutic effects. In order to solve these problems, we should use advanced drug analysis technology to identify specific chemical composition and the specific mechanism of action of traditional Chinese medicine.

## Perspectives

At present, the main methods for the treatment of colorectal cancer are surgery, radiotherapy and chemotherapy, but they have a variety of side effects, leading to poor prognosis of the patients. Traditional Chinese medicine could regulate intestinal flora to repair intestinal mucosal barrier and activate immune cells to restore normal immune function of the intestinal mucosa 28. In addition, traditional Chinese medicine could improve the sensitivity of anti-tumor drugs by regulating intestinal flora 27. Conversely, some ingredients of traditional Chinese medicine could not be directly absorbed and intestinal flora could promote the transformation of these ingredients 29.

Recent evidence has shown that gut microbiota could regulate the innate and/or the adaptive immune system and contribute to the development and metastasis of colorectal cancer 30-34. It is known that traditional Chinese medicine has detoxification and synergistic effects of immune modulation. Compared with simple radiotherapy and chemotherapy, the application of traditional Chinese medicine can effectively improve the patient's health, reduce the recurrence and metastasis rate of colorectal cancer and prolong the survival of patients compared with simple application of chemotherapy and radiotherapy. For patients who cannot tolerate surgery, radiotherapy and chemotherapy, the application of traditional Chinese medicine could be a promising strategy for the treatment of colorectal cancer.
